# Early Gray Matter Volume Loss in MAPT H1H1 *de Novo* PD Patients: A Possible Association With Cognitive Decline

**DOI:** 10.3389/fneur.2018.00394

**Published:** 2018-05-30

**Authors:** Frederic Sampedro, Juan Marín-Lahoz, Saul Martínez-Horta, Javier Pagonabarraga, Jaime Kulisevsky

**Affiliations:** ^1^Movement Disorders Unit, Neurology Department, Hospital de la Santa Creu i Sant Pau, Barcelona, Spain; ^2^Biomedical Research Institute (IIB-Sant Pau), Barcelona, Spain; ^3^Centro de Investigación Biomédica en Red Enfermedades Neurodegenerativas, Madrid, Spain

**Keywords:** MAPT, T1-MRI, cognitive decline, Parkinson's disease, gray matter volume

## Abstract

The MAPT H1 haplotype has been identified as a predictor of cognitive decline in Parkinson's disease (PD). However, its underlying pathological mechanisms have not been fully established. In this work, using a cohort of 120 *de novo* PD patients with preserved cognition from the Parkinson's Progression Markers Initiative (PPMI) database, we found that patients who were homozygous for MAPT H1 had less gray matter volume (GMV) and greater 1-year GMV loss than patients without this genetic profile. Importantly, these changes were associated with a longitudinal worsening of cognitive indicators. Our findings suggest that early GMV loss in MAPT H1H1 PD patients increases their risk to develop cognitive decline.

## Introduction

Cognitive decline is a core non-motor symptom of Parkinson's disease (PD). Given its high prevalence and negative impact on the patient's quality of life, identifying early prognostic markers of cognitive impairment in this population can help develop a person-centered care plan and achieve optimal clinical management (1).

Several genetic variants have been linked to cognitive impairment in PD. In particular, the MAPT H1 haplotype has been associated with an increased risk for the development of dementia in this population (2). The microtubule-associated protein tau (MAPT) gene is located on chromosome 17q21, where an inversion polymorphism of approximately 900 kb defines two haplotypes, H1 and H2. However, the underlying pathological mechanisms responsible for the association of this genetic variant and cognitive decline in PD remain poorly understood.

Here we hypothesize that, among recently diagnosed PD patients with preserved cognition, those with MAPT H1 homozygosis will exhibit a differential cross-sectional or longitudinal gray matter volume (GMV) pattern that could make them more prone to develop cognitive decline than patients without this genetic condition.

Using the data from the Parkinson's Progression Markers Initiative (PPMI), we aimed to validate our hypothesis using structural MRI and MAPT genotyping from *de-novo*, untreated and cognitively preserved PD patients. The PPMI is a large observational study that offers a superb framework to analyze cross-sectional and longitudinal biomarker interactions in early PD patients (3).

## Materials and methods

### Sample

A hundred and twenty PD patients were considered for this study. The inclusion criteria were: *de novo*, untreated, cognitively preserved [MoCA ≥ 26 (4)] and non-depressed [GDS-15 ≤ 7 (5)] PD patients who had a baseline T1-MRI and MAPT H1 haplotype status (rs17649553, which is in linkage disequilibrium with the H1 haplotype). We defined two groups of patients: those with MAPT H1 homozygosis (*N* = 79) and MAPT H2 carriers (*N* = 41). A 1-year follow-up T1-MRI scan was also available for 96 (64 MAPT H1H1) participants.

### Assessments

To isolate the possible effects of the MAPT H1 homozygosis on brain structure and cognitive decline, we considered the presence of other demographic, clinical, neuropsychological, and biological variables that could act as confounding factors known to affect brain structure and cognition (Table 1).

**Table 1:** Demographic, clinical, neuropsychological, and biomarker values for the two MAPT groups at baseline.

	**MAPT H1H1 (*n* = 79)**	**MAPT H1H2/H2H2 (*n* = 41)**	**Significance (*p*-value)**
Age [years]	60.1 ± 9.5	61.5 ± 9.9	0.45
Sex [Female/Male]	30/49	17/24	0.71 X^2^
Education [years]	15.35 ± 2.9	15.68 ± 2.9	0.57
Months since PD diagnosis	7.2 ± 7.7	6.5 ± 6.7	0.61
UPDRS-III	20.2 ± 9.2	19.9 ± 7.2	0.84
GDS-15	4.99 ± 1.1	5.1 ± 1.4	0.64
MoCA	28.3 ± 1.4	28.4 ± 1.2	0.67
BJLO	13.04 ± 2.1	12.6 ± 2.1	0.32
HVLT Delayed free recall	8.6 ± 2.8	9.5 ± 1.7	0.08
HVLT Total recall	25.2 ± 5.1	26.7 ± 4.3	0.09
Numbers and Letters	11.3 ± 2.5	10.5 ± 2.8	0.11
Semantic Fluency	21.6 ± 5.1	20.9 ± 4.7	0.47
SDMT	43.1 ± 9.8	41.4 ± 8.9	0.37
Phonetic Verbal Fluency	13.3 ± 4.4	12.6 ± 4.3	0.38
DATSCAN	1.3 ± 0.4	1.3 ± 0.4	0.73
CSF Aβ42 [pg/ml]	366.9 ± 93.9	370.3 ± 105.5	0.87
CSF tau [pg/ml]	44.7 ± 18.2	46.5 ± 18.8	0.61
CSF p-tau [pg/ml]	16.5 ± 10.2	16.9 ± 9.9	0.83
CSF α-Synuclein [pg/ml]	1774.7 ± 617.9	1927.5 ± 834.6	0.31
APOE4 (APOE4+/APOE4−)	24/46	10/27	0.44 X^2^

*To examine differences across groups, t-test analyses were used for continuous variables and X^2^ for categorical measures. UPDRS-III: total motor score for the Unified Parkinson's disease rating scale GDS-15: Geriatric Depression Scale (15-item), MoCA, Montreal Cognitive Assessment (MoCA) [total score]; BJLO, Benton Judgment of Line Orientation [total score]; HVLT, Hopkins Verbal Learning Test; SDMT, Symbol Digit Modality Test [total score]; DATSCAN, average striatal binding ratios of the caudate and putamen regions. BJLO, HVLT, DLY, HVLT SUM, LETNUM, SEMFLU, SDMT data were available for 119 participants. CSF Aβ42 and CSF α-Synuclein for 118 participants, CSF tau and p-tau for 116 participants, DATSCAN for 112 participants, and APOE4 status for 107 participants*.

We also investigated whether the MAPT haplotype was related to the longitudinal development of cognitive decline, which was defined as showing a MoCA score <26 (5) throughout a 4-year follow-up period.

Details about the considered clinical scales, genotyping, image acquisition, and other biological variables are available at http://www.ppmi-info.org/.

### T1-MRI neuroimaging methods

To study baseline gray matter volume (GMV) differences related to the MAPT H1H1 profile, we applied a standard GMV voxel-based morphometry (VBM) pipeline using SPM12 (http://www.fil.ion.ucl.ac.uk/spm/). T1-MRI scans were segmented and their associated GMV maps were then spatially normalized to the Montreal Neurological Institute (MNI) space using DARTEL. A Gaussian smoothing of 8 mm FWHM was applied to the resulting images.

To analyze differences in 1-year gray matter volume loss across groups, we used the SPM12 longitudinal VBM pipeline. Processing steps were pairwise registration, computation of Jacobian difference images (restricted to gray matter), and subsequent normalization to MNI space using DARTEL.

The resulting set of preprocessed GMV and GMV loss maps were entered into a voxelwise two-sample *t*-test analysis to study differential patterns related to MAPT H1 homozygosis. Age, sex, education, and total intracranial volume (TIV) were used as covariates of no interest. Results were considered significant at *p* < 0.005, uncorrected with a minimum cluster size of 50 voxels. Only cerebral regions in the MNI space with an a priori GM probability greater than 0.4 were considered.

Finally, aiming to study whether the obtained imaging results were related to cognitive progression, we correlated GMV and GMV-loss values at the identified clusters with the 4-year decrease in total MoCA score.

## Results

As seen in Table 1, no significant basal differences between MAPT groups were found (*p* < 0.05), ruling out the presence of confounding factors that could indirectly compromise brain integrity in the H1H1 group. Ninety-five percentage of the sample is Caucasian.

In terms of cognitive progression, we found that 58% of patients with MAPT H1 homozygosis developed cognitive decline over a 4-year follow-up period. This percentage was 36% for MAPT H2 carriers. Therefore, H1 homozygotes had an increased relative risk of 1.6 (CI 95%; 0.7–3.6) to develop cognitive decline with respect to H2 carriers. In this sample, the Delayed Recall and Visuospatial domains were the ones that became most commonly compromised in the set of patients that converted to cognitive impairment.

**Figure 1:**
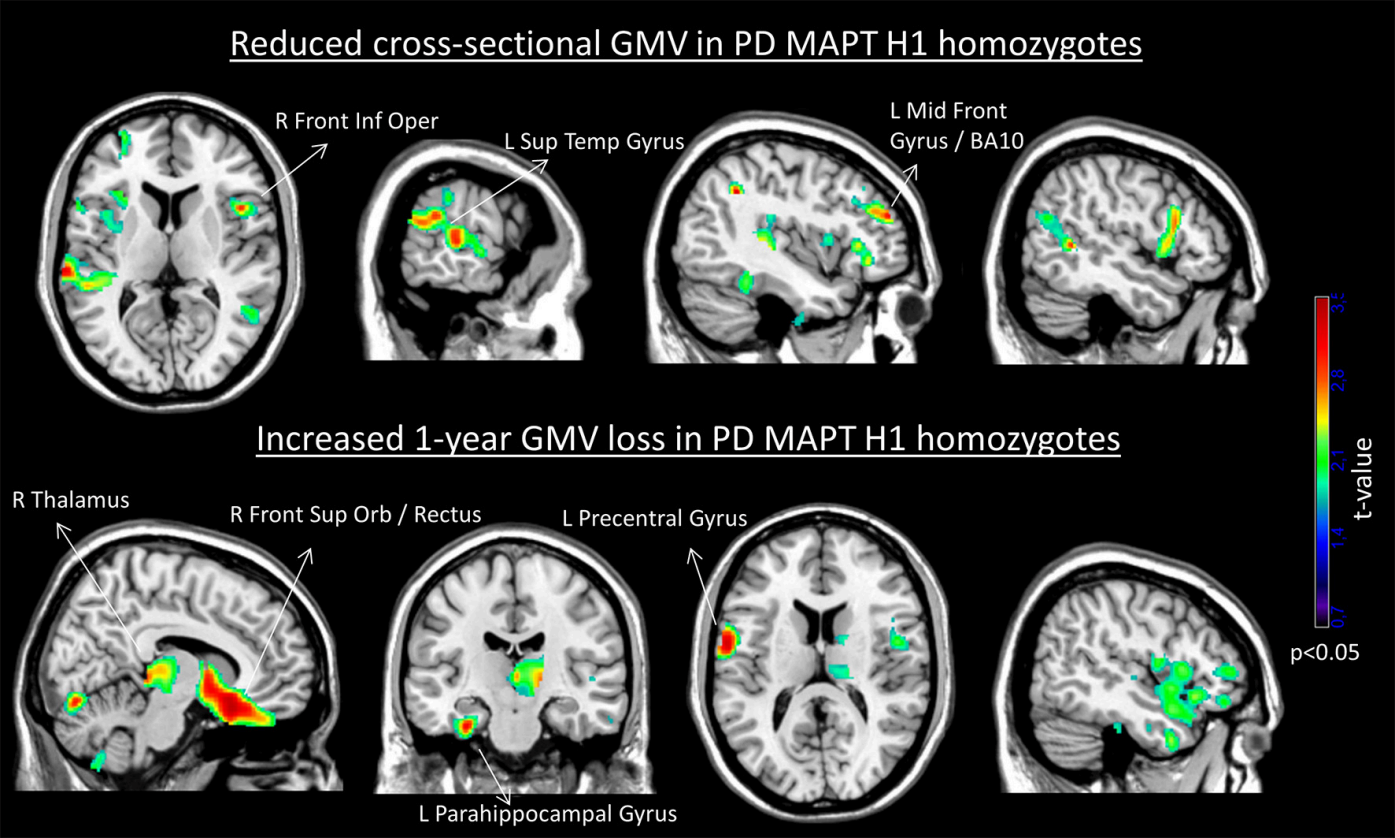
Cross-sectional reduced gray matter volume **(Top)** and increased 1-year GMV loss **(Bottom)** in the MAPT H1H1 group. For depiction purposes, results are displayed using *p* < 0.05 uncorrected and minimum cluster size (k) of 500 voxels. The set of labeled clusters survived a significance of *p* < 0.005 uncorrected and *k* = 50 voxels. No significant regions of increased gray matter volume or lower GMV loss were obtained in the H1H1 group.

Figure 1 shows the brain areas where MAPT H1 homozygotes showed cross-sectional and longitudinal gray matter volume differences with respect to the other genotypes.

Significant correlations were found between the GMV values at the identified clusters and the 4-year decrease in total MoCA score: left superior temporal GMV (*r* = −0.23, *p* = 0.025), left middle frontal gyrus GMV (*r* = −0.24, *p* = 0.021), right frontal inferior operculum GMV (*r* = −0.26, *p* = 0.014), and left parahippocampal GMV loss (*r* = 0.29, *p* = 0.011).

## Discussion

Here we investigated whether an early reduction in cerebral GMV could be a mechanism by which PD patients with MAPT H1 homozygozis have an increased risk to develop cognitive decline. In this group, we found a cross-sectional GMV reduction and an increased GMV loss over a 1-year period. Frontal and parieto-temporal areas were involved. These areas are known to play a major role in the emergence of mild cognitive impairment and its progression to dementia in PD (6).

The observed GMV compromise was associated in a unidirectional manner with the H1H1 group, without evidence of structural compensatory mechanisms. Importantly, GMV values in several of the obtained clusters were associated with a longitudinal decrease in MoCA scores.

Our results suggest that in PD, harboring the MAPT H1H1 genotype is associated with an early decrease in GMV, even in cognitively preserved patients, possibly making this population more prone to cognitive decline. A biological explanation for this observation could be related to an increased 4-repeat tau transcription associated with the H1 haplotype in PD brains, since this isoform has been suggested as the pathogenic driver (7, 8).

The effect of the MAPT H1 haplotype on brain function in PD patients and in control subjects has been addressed previously using functional MRI (9, 10). It was observed that subjects in the PD group and those in the control group with H1 homozygotes had an altered activation pattern while performing cognitive tasks. Furthermore, in line with our results, structural brain differences were found in healthy H1H1 subjects (11). Hence, our findings suggest that the potential underlying pathological mechanism of this genetic variant is shared across the spectrum of healthy subjects, individuals with Parkinson's disease, and people with other types of dementia (12).

Limitations of this work include the absence of a control sample and the use of MoCA scores to evaluate cognitive status. Even though this tool has shown some performance limitations in cognitive testing (4), it is commonly used in PD and is the only global and quantitative cognitive assessment available in PPMI. Additionally, in the light of the imaging results described here, further study into the particular relationship of this genetic variant and the worsening of specific cognitive domains and neuropsychological functions in PD could establish more specific clinical implications related to these findings.

To conclude, the present work reports a set of structural brain changes associated with MAPT H1 homozygosis in PD which may increase the risk of developing cognitive decline in this population. This observation sparks the need to further study the mechanisms by which tau transcription schemes could be responsible for a brain structural compromise in the early stages of PD.

## Author contributions

FS, JP, and JK conception or design of the work. FS, JM-L, and SM-H data analysis and interpretation. FS, JM-L, and SM-H drafting the article. JP and JK critical revision of the article.

### Conflict of interest statement

The authors declare that the research was conducted in the absence of any commercial or financial relationships that could be construed as a potential conflict of interest.
